# Influence of Vitamin C and Vitamin E on testicular zinc content and testicular toxicity in lead exposed albino rats

**DOI:** 10.1186/2050-6511-13-17

**Published:** 2012-12-14

**Authors:** Oluseyi C Ayinde, Sunday Ogunnowo, Rita A Ogedegbe

**Affiliations:** 1 Department of Biochemistry, College of Medicine, University of Lagos, Idi-Araba, Lagos, Nigeria

**Keywords:** Lead, Zinc, Vitamin C, Vitamin E, Oxidative stress, Testicular toxicity

## Abstract

**Background:**

Occupational and environmental exposures to lead remain a public health problem as lead alters physiological processes by inducing oxidative stress and mimicking divalent cations. This study was designed to investigate the effects of Vitamin C (VC) and Vitamin E (VE) on the reproductive function of lead exposed male rats. Experimental animals were exposed to oral doses of lead, VC and VE at 60 mg/kg body weight, 40 mg/kg body weight, and 150 mg/kg body weight respectively, while control animals received 0.9% saline solution. Oral administration spanned for six weeks after which changes in testicular redox status, lead deposition, testicular zinc content, serum androgen content, semen quality and testis histology were examined.

**Results:**

There were significant (p < 0.05) increases in oxidative stress indices and testicular lead content. A significant (p < 0.05) depletion of zinc in the testis of lead exposed animals was also observed. Fluctuations were observed in androgen levels of lead treated animals with a significant increase (p < 0.05) in Serum follicle stimulating hormone (FSH) and testosterone (TT) content, while there was no significant change in luteinizing hormone (LH) content. Testicular tissue showed an alteration in its normal histology with degeneration of the seminiferous epithelium accompanied by a significant reduction (p < 0.05) in the number of luminal spermatozoa. A downgrade in the semen appearance and semen quality –sperm motility, morphology, and count was also observed after lead exposure. VC and VE treatment showed a significant (p < 0.05) reversal of the physiological alteration induced by lead.

**Conclusions:**

Lead exposure resulted in a decline in the reproductive function of male rats by inducing oxidative stress, inhibiting enzymes and depleting testicular zinc contents. However, results clearly showed that VC and VE attenuated the deleterious impact of lead on the reproductive system.

## Background

Lead poisoning is an age long environmental hazard [1]. Industries account for an annual production of about 2.5 million tons of lead globally [2]. Lead is present in batteries, leaded gasoline [3], paints, water pipes, insecticides, and some cosmetics. Air, water, soil, food and consumer products are the major routes of human exposure to lead [4]. The accumulation of lead in various tissues and its interference with bioelements accounts for its pathophysiology [5]. The toxic effect of lead on reproduction is pervasive affecting basically all aspects of the reproductive system [6]. One mechanism by which lead exerts its adverse effect is by inducing oxidative stress (OS). Oxidative stress represents an imbalance between the production of reactive oxygen species (ROS) and a biological system's antioxidant defence mechanism [7]. Because of a high content of polyunsaturated membrane lipids testicular tissue becomes one of the targets for OS [8].

Zinc (Zn) plays an important role in the reproductive system [9]. It is the only metal found in almost all classes of enzymes. High concentrations of zinc found in the testes and accessory sex glands show its pivotal role in the reproductive system [10]. Zn deficiency has been linked to hypogonadism [11] and impaired sperm function [12].

Studies have shown that antioxidants have a far-reaching effect in andrology [13]. Vitamins A, E, D and C were reported to possess antioxidant functions, and their inhibitory effects on ROS accumulation vary from one vitamin to the other [14]. L-Ascorbic acid (Vitamin C) is a water soluble vitamin derived from dietary sources such as fruits and vegetables [15]. Vitamin C (VC) plays an efficient protective role directly or indirectly in a systemic detoxification of Pb [16]. Vitamin E (VE) a term that encompasses a group of potent, lipid-soluble, chain breaking antioxidants [11] which acts as a naturally occurring antioxidant. VE locates itself in biological membranes where it functions to inhibit oxidation and oxidative damage to membrane polyunsaturated fatty acid (PUFA) [17].

Since the environment remains an integral portion of human existence coupled with lead’s pervasiveness within the environment, it has become imperative to find out inexpensive and simple ways by which the body can be made to maintain homeostasis after lead exposure. This work, therefore, aims at finding out whether or not oral administration of VC and VE either individually or in combination would proffer any form of ameliorative effect on testicular toxicity, zinc concentration and testicular lead burden in lead exposed rats.

## Results

Data in Table 1 show that rats exposed to lead exhibited a significant decrease in the glutathione content, superoxide dismutase and catalase enzyme activity in the testis when compared with the control group. More so, lead treatment increased significantly the amount of lipid peroxidation products (MDA), total protein and nitric oxide content in the testis. However vitamin C and/or vitamin E treatment significantly increased glutathione content, superoxide dismutase and catalase enzyme activity, while also decreasing lipid peroxidation products, total protein and nitric oxide contents when compared to the lead only group.

**Table 1:** Oxidative stress markers of albino rats

**Parameter**	**Control**	**Pb**	**Pb + VC**	**Pb + VE**	**Pb + VC + VE**
**GSH (U/mg Protein)**	0.69 ± 0.04	0.10 ± 0.02 ^a^	0.51 ± 0.03 ^ab^	0.52 ± 0.03 ^ab^	0.57 ± 0.02 ^ab^
**SOD (U/mg Protein)**	9.19 ± 0.69	1.20 ± 0.17 ^a^	5.40 ± 0.43 ^ab^	6.04 ± 0.55 ^ab^	6.17 ± 0.07 ^ab^
**CAT (U/mg Protein)**	42.48 ± 2.37	9.48 ± 0.36 ^a^	28.90 ± 0.78 ^ab^	26.44 ± 1.33 ^ab^	31.30 ± 0.69 ^ab^
**MDA (Mmol/g tissue)**	0.35 ± 0.02	0.94 ± 0.03 ^a^	0.44 ± 0.02 ^ab^	0.47 ± 0.02 ^ab^	0.43 ± 0.01 ^ab^
**PRT (mg/g tissue)**	20.36 ± 0.71	30.29 ± 1.75 ^a^	21.54 ± 1.55 ^b^	18.69 ± 1.71 ^b^	21.00 ± 1.04 ^b^
**NO (mg/dL)**	15.84 ± .86	42.12 ± 1.70 ^a^	25.42 ± 1.65 ^ab^	26.98 ± 1.60 ^ab^	22.96 ± 1.24 ^ab^

Values are expressed as Mean ± S. E. M.

a: Significant with control.

b: Significant with lead only group.

c: Significant with Vitamin C group.

d:Significant with Vitamin E group.

Significant at p < 0.05.

The number of animals in each experimental group was 5.

Data in Table 2 reveal that lead treatment resulted in a significant increase in lead accumulation in the testis of lead exposed animals, with a concomitant decrease in zinc content when compared to the control. Nevertheless, vitamin C and/or E treatment reduced lead content and increased zinc content in the testes of lead treated animals when compared with lead only group.

**Table 2:** Metal contents in the testes of albino rats

**Metals**	**Control**	**Pb**	**Pb + VC**	**Pb + VE**	**Pb + VE + VC**
**LEAD (mg/g tissue)**	0.0245 ± 0.002	1.256 ± 0.008 ^a^	0.586 ± 0.048 ^ab^	0.840 ± 0.041 ^abc^	0.521 ± 0.011 ^abd^
**ZINC (mg/g tissue)**	0.541 ± 0.014	0.107 ± 0.012 ^a^	0.233 ± 0.009 ^ab^	0.1401 ± 0.007 ^ac^	0.281 ± 0.007 ^abcd^

Values are expressed as Mean ± S. E. M.

a: Significant with control.

b: Significant with lead only group.

c: Significant with Vitamin C group.

d:Significant with Vitamin E group.

Significant at p < 0.05.

The number of animals in each experimental group was 5.

Table 3 shows that lead treatment accounted for a significant decrease in serum follicle stimulating hormone and testosterone of lead treated rats. However, no significant decrease was observed in the luteinising hormone content when compared to the control. Vitamin C and/or vitamin E treatment increased significantly the contents of follicle stimulating hormone, and testosterone content in the serum of lead treated rats when compared to the lead alone group.

**Table 3:** Hormone profile in the blood sera of albino rats

**Hormone**	**Control**	**Pb**	**Pb + VC**	**Pb + VE**	**Pb + VE + VC**
**LH(mIU/L)**	1.02 ± 0.05	0.46 ± 0.10	0.86 ± 0.08	1.1 ± 0.26 ^b^	1.04 ± 0.10
**FSH(mIU/L)**	1.68 ± 0.12	0.68 ± 0.11 ^a^	1.46 ± 0.05 ^b^	1.16 ± 0.05 ^ab^	1.44 ± 0.08 ^b^
**TT(nmol/L)**	39.00 ± 1.18	9.64 ± 0.52 ^a^	35.60 ± 1.03 ^b^	21.80 ± 1.16 ^abc^	36.40 ± 0.93 ^bd^

Values are expressed as Mean ± S. E. M.

a: Significant with control.

b: Significant with lead only group.

c: Significant with Vitamin C group.

d:Significant with Vitamin E group.

Significant at p < 0.05.

The number of animals in each experimental group was 5.

Table 4 shows that lead exposed animals had a significant decrease in the percentage of sperm motility and sperm count when compared with the control group. They also had an increase in abnormal sperm morphology. However vitamin C and/or vitamin E treatment significantly increased sperm motility and count in lead treated animals, and also reduced the percentage of abnormal sperm morphology in lead exposed animals, when compared to the lead alone group

**Table 4:** Status of spermatozoa in albino rats

**Parameter**	**Control**	**Pb**	**Pb + VC**	**Pb + VE**	**Pb + VE + VC**
**MOTILITY(%)**	46.40 ± 1.21	28.20 ± 1.28 ^a^	35.20 ± 0.86 ^ab^	37.60 ± 0.93 ^ab^	43.60 ± 1.03 ^bcd^
**COUNT(sperm x10**^**6**^**/ml)**	53.75 ± 2.24	36.00 ± 1.28 ^a^	44.25 ± 2.04 ^ab^	41.25 ± 1.63 ^a^	45.55 ± 2.18^ab^
**MORPHOLOGY CHANGE (%)**	6.00 ± 0.95	21.00 ± 1.38 ^a^	8.80 ± 0.37 ^ab^	11.60 ± 0.75 ^ab^	7.80 ± 0.5 ^bd^

Values are expressed as Mean ± S. E. M.

a: Significant with control.

b: Significant with lead only group.

c: Significant with Vitamin C group.

d:Significant with Vitamin E group.

Significant at p < 0.05.

The number of animals in each experimental group was 5.

**Figure 1:**
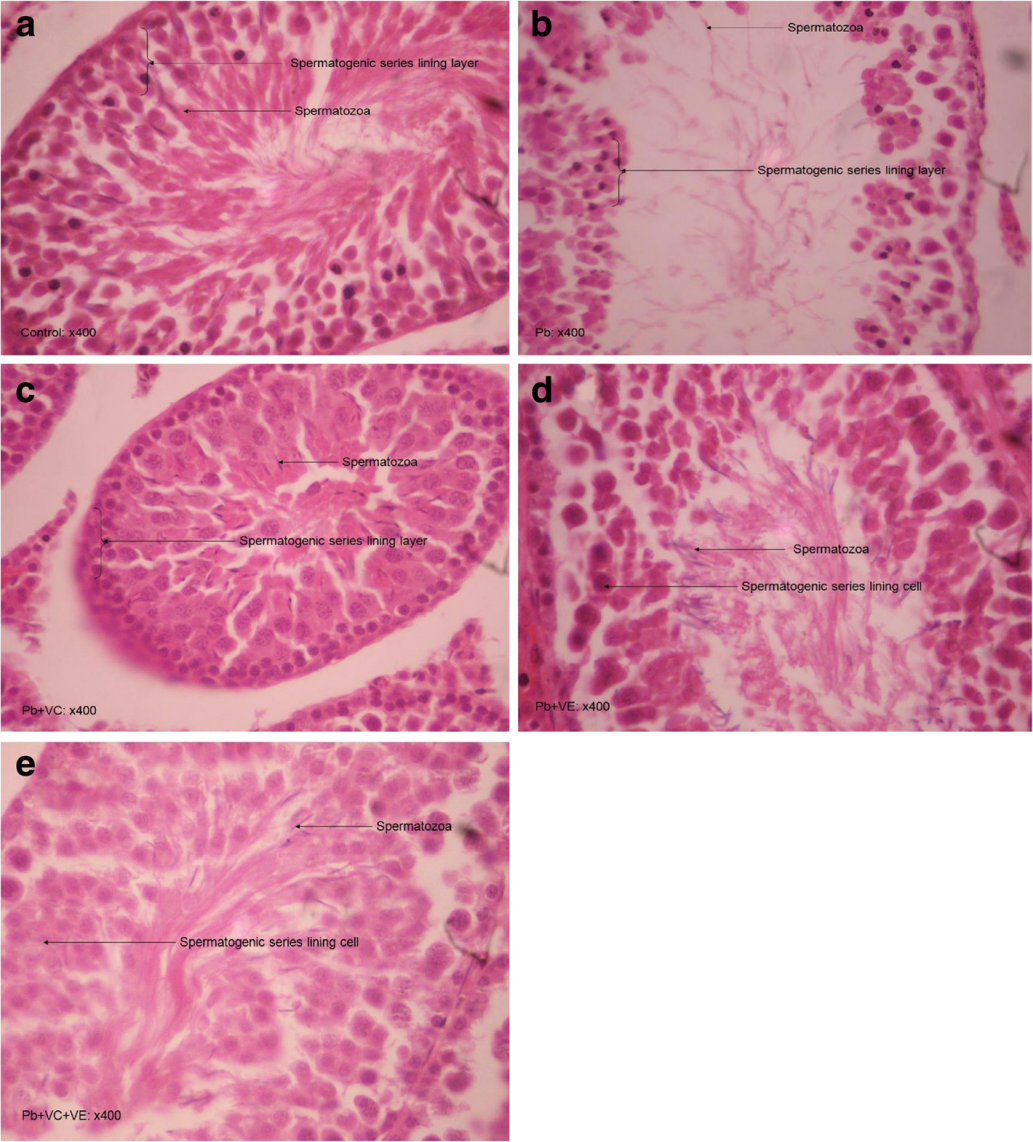
**Histological changes in testes of rats (magnification x400) (spermatogenic series and spermatozoa illustrated).** (**a**). Control testes showing normal* cell histology. (**b**). Pb only group- testes showing Mild reduction in seminiferous epithelium, empty lumen and reduction in number luminal spermatozoa in areas. (**c**). Pb + VC group-testes showing normal* cell histology. (**d**). Pb + VE group-testes showing an increase in luminal spermatozoa. (**e**). Pb + VC + VE group- Increase in luminal spermatozoa. *Normal: Seminiferous tubules contain 3–5 cell layered spermatogenic epithelium, with moderate amount of luminal spermatozoa.

Figure 1 presents the histological changes in lead exposed animals, lead exposed animals had a reduction in the seminiferous epithelium accompanied with a reduction in the lumina spermatozoa when compared to control group. However improvements were observed with vitamin C and/or E treatment.

## Discussion

Occupational and environmental exposures to heavy metals are known to have deleterious effects on human reproduction. In rats lead (Pb) has been implicated in male reproductive dysfunction [18]. In this study our results clearly demonstrates that lead acetate exposure can seriously alter the testes and reproductive function of adult male rats. Oxidative stress parameters were examined in testicular homogenate, and lipid peroxidation as measured by the amount of MDA was significantly (p < 0.05) elevated in the testes of lead treated rats (Table 1). Lipid peroxidation has been implicated in the aetiology of damage to subcellular membranes by inactivating cell constituents through oxidation or causing oxidative stress by undergoing radical chain reaction [19]. The stimulation of lipid peroxidation, seemingly caused by lead treatment is related to the formation of free radicals after depletion of antioxidants. Table 1, shows a significant (p < 0.05) decline in the production of glutathione in the testes of lead exposed animals, as well as lead alteration in the function of some antioxidant enzymes markers. Lead exposure resulted in a significant (p < 0.05) decrease in superoxide dismutase and catalase enzyme activities in the testes of the rats. This observations are similar to those made by Salawu *et al.,*[20]. Our study also showed that there was a significant (p < 0.05) increase in the levels of nitric oxide content in the testes of the animals similar to observations made by Moniem *et al.,*[6]. All these culminate to a depletion of total antioxidant content and an increase in reactive oxidant specie. Vigeh *et al.,*[21] describes lead’s ability to elevate ROS in biological systems as correlated to its active inhibition of sulfhydryl antioxidants synthesis, inhibition of enzyme reactions, damage to nucleic acids and inhibition of DNA repair. As such our results indicate that lead acetate induced an embellished inhibitory effect on GSH, SOD and CAT.

Testicular protein content was significantly (p < 0.05) increased in lead treated animals. Afaf *et al.,*[22], hypothesised that these changes in protein content could result from reduction in the synthetic activity in testes, since testicular fluid contains both stimulatory factors and inhibitory factors that alter protein secretions [23]. Furthermore, Afaf *et al.,*[24] suggested that protein accumulation in the testes could be due to androgen deprivation.

Zinc is essential in spermatogenesis, a cofactor of metalloenzymes, involved in DNA transcription, involved in steroid receptor expression, and protein synthesis [25]. Results in Table 2 showed that lead treated animals showed a significant (p < 0.05) decrease in testicular zinc content. Victery *et al.,*[26] reported a similar reduction in zinc content in the plasma and testes of lead exposed male rats. They further revealed that lead’s ability to significantly increase urinary excretion of zinc was a possible mechanism for loss of testicular zinc. More so, another mechanism in which Pb exerts its toxicity is by substituting Zn in zinc-mediated processes [27]. This trend could also explain the lead induced SOD-enzyme inhibitory effect as lead replaces Zn in its sites. Such interaction has been demonstrated where lead replaces Zn to form Cu-Pb-SOD [14]. Our studies revealed that lead treated animals also showed an elevation in testicular tissue lead content. Therefore a possible relationship could be drawn between lead exposure and zinc depletion in the testes of albino rats.

Results in Table 2 also show that vitamin C and vitamin E treatment either individually, or in combination ameliorated the inhibitory action of lead by removing ROS once formed. Interestingly, Vitamin C treatment significantly (p < 0.05) reduced the testicular lead content and increased the Zinc contents in the lead treated rats when compared to vitamin E treatment. The biochemical parameters considered in this study showed a significant increase in total antioxidant content, and a reduction in lipid peroxidation products in vitamin C and/or vitamin E treatment (Tables 1 and 2).

Table 3 showed that serum FSH and TT levels in the animals were altered in the lead exposed animals, this was similar to the study of Ait *et al.,*[28]. LH results showed no significant alteration in lead exposed animals when compared to the control group. This is in agreement with El-tohamy [29] who observed that lead suppressed TT production with no significant alteration to LH content. However, studies carried out by Salawu *et al.,*[20] showed no significant decrease in serum TT level in lead treated rats. Nevertheless, reduction of LH and TT levels in animals exposed to lead indicates enzyme inhibition in the steroidogenetic pathway [30]. More so, increased blood lead content has been associated with disruption of hypothalamic secretion of hormones and spermatogenesis [28].

The testicular sperm counts and daily sperm production are important indicators of spermatogenesis [31]. In this study (Table 4), administration of lead acetate resulted in an obvious decline in sperm density, significantly (p < 0.05) reducing sperm counts, sperm motility, and increasing the amount of anomalous spermatozoa, [32]. OS is associated with sperm function impairment and plays a major role in the aetiology of defective sperm function [14]. Lead’s direct toxicity on testicular histology or its action on the hypothalamic-pituitary axis or a combined defect involving the gonad and hypothalamic-pituitary sites could inhibit spermatogenesis [33]. Zinc deficiency has also been reported to affect sperm membrane integrity, and to reduce sperm motility. A possible correlation could also be drawn from lead induced zinc deficiency and sperm quality in rats.

Nonetheless vitamin C and/or vitamin E treatment on lead intoxicated animals showed a significant increase in reproductive hormones and an ameliorative effect on the semen quality assessed. Vitamin C treatment showed a higher ameliorative effect on testosterone, follicle stimulating hormone levels, and sperm motility and morphology when compared to vitamin E treatment.

Histological examination in Figure 1 shows deformities in the testicular tissue architecture of lead treated animals, with serious damages within the semniferous tubules. Lead treated animals showed reduction in seminiferous epithelium, an empty lumen with concomitant reduction in the number luminal spermatozoa. These findings are in agreement with Moniem *et al.,*[6] whose work showed that lead exposure caused progressive vascular, tubular and interstitial testicular damage. One possible mechanism for this impairment could be attributed to zinc reduction, as zinc deficiency in rats have been suggested to result in atrophy of seminiferous tubules [10]. More so lead induced OS could also cause oxidative damage to cellular materials.

Treatment of lead exposed animals with vitamin C and/or vitamin E showed an increase in luminal spermatozoa and seminiferous epithelium, with VC treatment potentiating better effects as viewed in the tissue histology. Zhu *et al.,*[34] reported a similar increase in the density of semineferous cells and luminal spermatozoa in Boer goats supplemented with vitamin E.

The ability for Vitamin C to exhibit ameliorative effect stems from its antioxidant, and chelating properties. In previous studies administration of ascorbic acid decreased significantly but not completely Pb circulation in the blood. Some other studies in rats demonstrated that ascorbic acid decreased the intestinal absorption of lead [35] and increased the renal clearance of lead [36]. VC has also been reported to protect the cells and sperm from oxidative stress and loss of motility, respectively [37]. VC inhibits lipid peroxidation, regenerate spent VE and protects against hydrogen peroxide induced DNA damage [15].

More so, VE was found to exhibit a protective effect on the testis of rats. It is a major chain-breaking antioxidant in the sperm membranes. It acts as a free radical scavenger, scavenging superoxide, hydrogen peroxide, and hydroxyl radicals [13]. Adding VE in diet increased the activity of some antioxidant enzymes, decreased nitric oxide content and lipid peroxidation products in the testis of Boer goat [34].

Vitamin C and E have both been implicated in protecting sperm DNA from oxidative stress of free radicals and improving fertility [38]. The differential in the ameliorative effect of Vitamin C and Vitamin E on the Pb-exposed animals could be related to the ability of vitamin C to chelate lead, thereby reducing leads capacity to substitute zinc in zinc mediated processes, recycling vitamin E and also directly participating in antioxidant activity by helping to replenish glutathione.

## Conclusions

In conclusion, the present study showed that oral administration of lead was responsible for histological damage and disturbances in the metabolism of male reproductive organs. Nonetheless ameliorative effects where observed with vitamin C and/or Vitamin E treatment, with vitamin C potentiating a better ameliorative effect than vitamin E. Vitamin C and E proved to be ameliorative by improving the redox status, replenishing testes zinc content, reducing lead burden, reverting androgen levels, semen quality and testicular histology towards normal physiology.

## Methods

### Animals

Twenty five sexually matured, 12 weeks old albino Sprague Dawley Male rats (120-160 g) were obtained from the animal house of the College of Medicine, University of Lagos, Idi-araba Lagos state, Nigeria. This research was conducted in accordance with the internationally accepted principles for laboratory animal use and care as found in the US guidelines (NIH publication # 85–23, 1985) and approved by the committee on ethics in animal experimentation of the college of medicine, university of Lagos, Lagos Nigeria. The animals were kept in plastic cages and maintained under standard animal house conditions of illumination (illuminated for 12 h per day 0700-1900 h), and ventilation (kept in a room with a temperature of 28 ± 2°C). They had access to standard rat chow and water *ad libitum*.

### Experimental Design

The animals were randomly and equally divided into five (5) groups. The rats were housed 5 in a plastic cage, and acclimatized for 14 days. After 14 days of acclimatization, a sub chronic study of six weeks was conducted. Lead was administered at 60 mg/kg body weight as lead acetate; Ascorbic acid was administered at 40 mg/Kg body weight as L ascorbic acid (VC), and α-tocopherol (VE) at 150 mg/Kg body weight as α-tocopheryl acetate. The first group served as control and received normal saline (0.9% w/v NaCl), the second group served as the lead (Pb) only group and received lead only, the third group served as the lead and L ascorbic acid (Pb + VC) group and received Pb and twenty minutes later VC. The fourth group served as the lead and α- tocopheryl acetate (Pb + VE) group and received Pb and twenty minutes later VE. The fifth group served as the lead and L ascorbic acid and α-tocopheryl acetate (Pb + VC + VE) group and received Pb and twenty minutes later L ascorbic acid and α-tocopheryl acetate.

The administration, of saline, lead, L ascorbic acid, and α-tocopheryl acetate was done three (3) times a week, every other day. All administrations were done via oral route.

### Laboratory procedures and sample collection

At the end of the six weeks exposure and consequently after 24 h of the last oral administration, blood samples were collected via the orbital plexus into a standard test tubes from all animals and the animals were sacrificed by cervical dislocation. Blood samples stood for half an hour and then centrifuged at 500x g for 15 mins at 4°C to separate serum and stored at −70°C.Also the testes were quickly excised suspended in cold normal saline to rinse, weighed and kept cold for homogenisation.

### Histological examination

After sacrificing rats in each group, the testis was quickly removed. The tissue was immersed in 10% formal saline for 16 hr for tissue fixation. Afterwards it was rinsed with distilled water, dehydrated in graded alcohol, cleared in xylene, and embedded in paraffin. Finally, they were cut into 5 μm section with a rotary microtome and stained with haematoxylin and eosin and examined under a microscope [39].

### Biochemical indices

#### Determination of serum hormones

Serum testosterone (TT), follicle stimulating hormone (FSH) and luteinizing hormones (LH) where quantitatively measured by adopting enzyme-linked immunosorbent assay (ELISA) technique using kits purchased from Rapid Labs Ltd., (Essex, U.K).

#### Determination of lipid peroxidation products (MDA)

Malondialdehyde, formed from the breakdown of polyunsaturated fatty acids, serve as a convenient index for determining the extent of lipid peroxidation that reacts with thiobarbituric acid to give a red specie absorbing at 535 nm [40].

#### Determination of reduced gluthatione content (GSH)

Determination of reduced glutathione is based on the formation of a yellow colour after reacting with 5,5'dithiobis-2-nitrobenzoic acid (DTNB) which is then read at 412 nm [41].

#### Determination of super oxide dismutase (SOD) activity

SOD activity was determined according to the method by Sum and Zigma, [42]. Here the SOD enzyme assay is determined by the difference between superoxide anion decomposition and production i.e. its ability to inhibit the autoxidation of epinephrine, determined by the increase in absorbance at 320 nm.

#### Determination of catalase enzyme activity (CAT)

The activity of the enzyme catalase was analysed according to Cohen *et al.,*[43], which measures the initial rate of hydrogen peroxide (50 mM) decomposition. Where one unit is the amount of enzyme that hydrolyses 1 mol of H2O2 per minute and per milligram of protein at 25°C and pH 8.0.

##### Determination of total protein content (PRT)

This method is based on the reaction between the tryptophan and tyrosine residues of the protein and the phosphomolybdic - phosphotungestic acid reagent (Folin Ciocalteu's reagent) in alkaline copper solution. The developed blue colour was then measured colouremetrically at a wave-length of 690 nm [44].

#### Sperm analysis and evaluation

Sperm samples obtained from the distal region of the vas deferens of the rats were used. This involved excision of a small piece of the vas deferens just distal to the cauda epididymis. The excised pieces were placed in a Petri dish containing 4 ml of normal saline (0.9% sodium chloride) at 37°C and homogenized. Sperm motility, count and morphology were determined through microscopic examination [45].

#### Metal analysis

Testes were digested with concentrated nitric acid and digests were diluted to a constant volume with deionised water. Lead and zinc concentration where then determined from the resultant solution by atomic absorption spectrophotometry (AAS).

#### Statistical analysis

Results were presented as Mean± S.E.M. Statistical analysis was conducted using one way ANOVA to compare the means and Tukey’s test for post hoc. P-value <0.05 was considered significant. Statistical analysis was done using SPSS® 16 statistical software for windows.

## Abbreviations

Pb: Lead; VC: Vitamin C; VE: Vitamin E; ROS: Reactive Oxygen Species; OS: Oxidative Stress; GSH: Glutathine; SOD: Superoxide Dismutase; CAT: Catalase; MDA: Malondialdehyde; NO: Nitric Oxide; TT: Testosterone; LH: Luteinising hormone; FSH: Follicle stimulating hormone; PRT: Total protein content; Zn: Zinc.

## Competing interests

The authors declare that they have no competing interests.

## Authors’ contributions

RAO was involved in the histological examination, and participated in animal care. SO carried out the immunoassays. OCA conceived of the study, and participated in its design and coordination, OCA participated in animal care, participated in enzyme assay and metal analysis and drafted the manuscript. All authors read and approved the final manuscript.

## Pre-publication history

The pre-publication history for this paper can be accessed here:

http://www.biomedcentral.com/2050-6511/13/17/prepub
